# Alzheimer‐related neuropathology is improved by treatment with longevity‐enhancing treatments in aged male EFAD mice

**DOI:** 10.1002/alz70855_099102

**Published:** 2025-12-23

**Authors:** Amy Christensen, Cassandra J McGill, Ali Zaidi, Christian J. Pike

**Affiliations:** ^1^ University of Southern California, Los Angeles, CA, USA

## Abstract

**Background:**

The greatest risk factor for Alzheimer's disease (AD) is aging. The e4 allele of apolipoprotein E (*APOE4*) is the primary genetic risk factor for AD and is associated with decreased longevity in both humans and rodents. Many of the pathways disrupted during aging are also (i) impacted *APOE4* genotype, and (ii) implicated in AD pathogenesis, suggesting that therapeutics known to improve health‐ and lifespan may be repurposed to function as effective interventions for AD, especially in the context of *APOE4*.

**Method:**

To investigate this topic, we studied the independent and combined efficacies of two longevity treatments in EFAD mice, a rodent model with knock‐in of human *APOE3* or *APOE4* crossed to the 5xFAD AD mouse. The first intervention was fasting mimicking diet (FMD), a version of caloric restriction that cyclically (repeated for 4 consecutive days every 2 weeks) reduces calories while maintaining micronutrients intake. The second intervention was 17α‐estradiol (17αE2), a naturally occurring low potency estrogen and diastereomer of the primary estrogen, 17β‐estradiol. 17αE2 has been shown to increase lifespan as well as beneficially regulate inflammatory and metabolic pathways in male mice. Sixteen‐month‐old male *APOE3* and *APOE4* EFAD mice were randomized into one of four treatment groups: (1) vehicle + *ad libitum* diet; (2) vehicle + FMD; (3) 17αE2 + *ad libitum* diet; or (4) 17αE2 + FMD. After nine weeks of treatment, a range of systemic and neural outcomes were tested.

**Result:**

Both 17αE2 and FMD independently reduced AD neuropathology in the EFAD mice, in an *APOE*‐dependent manner. Protective effects of both treatments were strongest in mice with *APOE4* genotype. Combining 17αE2 and FMD did not result in additive benefits.

**Conclusion:**

Longevity‐enhancing interventions have significant potential as AD therapeutics, even when administered as treatment at late stages of pathogenesis. Continued study and refinement of these highly translatable interventions are warranted.

